# Costs of non-multidrug-resistant pulmonary tuberculosis and of preventive treatment in Germany – An update

**DOI:** 10.1016/j.jctube.2024.100473

**Published:** 2024-07-26

**Authors:** Roland Diel, Albert Nienhaus

**Affiliations:** a Institute for Epidemiology, University Medical Hospital Schleswig-Holstein, Kiel, Germany; b Lung Clinic Grosshansdorf, Airway Research Center North (ARCN), German Center for Lung Research (DZL), Germany; c Institute for Health Service Research in Dermatology and Nursing (IVDP), University Medical Center Hamburg-Eppendorf, Hamburg, Germany; d Institution for Statutory Accident Insurance and Prevention in the Health and Welfare Services (BGW), Hamburg, Germany Start, Germany

**Keywords:** Cost, Tuberculosis, Contact investigations, Preventive treatment, Public Health

## Abstract

**Background:**

Only 4076 new cases of tuberculosis (TB) were reported in Germany in 2022; of those 184 were multidrug-resistant TB (MDR-/RR-TB).

**Methods:**

Based on the current therapy guidelines of the German Central Committee against Tuberculosis and most recent renumeration data of the Statutory Health Insurances (SHI), this study estimates the mean in- and outpatient costs per adult infectious pulmonary non-MDR-TB patient, together with costs arising from Rifampicin (RIF)-based short-course options of tuberculosis preventive treatment (TPT) of their close contacts.

**Results:**

From the insurance perspective, the mean inpatient cost (rounded) per adult case was 6138 EUR (SD±2810 EUR) for standard therapy; the cost of primary outpatient treatment only amounted to 1930 EUR and the cost of outpatient treatment post-hospital to 1093 EUR. The mean weighted cost was 6377 EUR (SD±2357 EUR), a drop of 27 % vs. 2019. This is mainly due to a decrease in hospitalizations of 5.6 %, and, given hospital treatment, by a 95 EUR decrease in the per-day reimbursement rate for TB patients who are hospitalized for at least 14 days. In contrast, the mean costs of TPT per person were 466 EUR (RIF solely over 4 months) and 423 EUR (RIF combined with Isoniazid over 3 months).

**Conclusion:**

While costs for active non-MDR-TB treatment in Germany have clearly decreased thanks to increased engagement on the part of the private practice sector and lower reimbursement rates in hospital, the comparatively high costs of short-course TPT have surprisingly significant economic impact. This negative development can be countered through diligent selection of close contacts persons of infectious TB cases before using IGRA testing to detect latent TB, to minimize the number of those persons who are tested falsely determined to be at risk and needlessly undergo TPT.

## 1 Introduction

The status of tuberculosis (TB) as one of the deadliest human infectious diseases has in recent years been upheld, despite the spectacular rise of coronavirus disease 2019 (COVID-19). Whereas COVID-19 has caused nearly 7 million deaths since its widespread emergence in 2020 [1], about 1.6 million people died from TB during that year alone, according to the World Health Organization (WHO)’s 2022 Global TB Report [2]. Furthermore, recent evidence suggests that TB infection increases a patient’s vulnerability to COVID-19, as well as their likelihood of dying from the disease or enduring a prolonged recovery [1]. The rapid diagnosis and treatment of active TB has therefore become an even more urgent concern, as has establishing TPT for latently infected contacts of infectious pulmonary TB patients. The latter measure is aimed, of course, at breaking the infection chain, thus reducing the number of subsequent TB cases to be expected in the future. This analysis examines the current reimbursement situation for treatment of pulmonary tuberculosis in Germany and compares its costs with those of RIF-based regimen for TPT. The combination of Isoniazid (INH) and RIF daily for 3 months or a RIF monotherapy for 4 months showed no inferiority in *meta*-analyses compared to at least six months of daily INH, but better completion rates. Hence these shorter regimens are those preferred in the latest recommendations of the German Central Committee against tuberculosis (DZK) [3], [4].

## 2 Epidemiological background

Since 2018, the number of TB cases reported to the Robert Koch Institute for Germany has fallen substantially. Versus 5429 cases reported for 2018 [5], the number for 2022 was 4076, a drop of 1353, or 24.9 %, in just four years. This gives our country a TB incidence rate of only 4.9 cases per 100,000 population [6]. Only 190 new cases were reported in children, meaning that the great majority of cases (95.3 %) relates to adults (aged 15  years or older). Cases treated in hospital wards and followed up by physicians in private practice were in 2022 only 83.9 % of the total, compared to 89.5 % in 2018, thus continuing the downward trend of recent years [6]. MDR-/RR-TB was determined in 184 cases.

## 3 Methods

Our cost calculations are based on the most recent renumeration data of the German SHI and on the procedures and therapeutic suggestions stated explicitly or directly derived from the new guidelines of the DZK [3], [4]. The associated costs for reimbursing doctors and laboratories in private practice can be derived from the Uniform Appraisal Scale (EBM, *Einheitlicher Bewertungsmaßstab*) [7]. Costs of hospital treatment effectively incurred by the SHI are established by the Institute for the Hospital Remuneration System (InEK, *Institut für das Entgeltsystem im Krankenhaus*) [8] for the German inpatient-DRG system which allocates each hospital case to a diagnosis-related group. For TB, there are three DRG categories; the costs for E76B cases (TB with severe complications, hospital stay less than 14 days) and E76C cases (TB without severe complications, hospital stay less than 14 days) are set by multiplying the national base rate (“Bundesbasisfallwert”) for the respective year with different cost weights, depending on the particular degree of disease severity, to arrive at a fixed level of reimbursement.

For TB patients being treated in hospital for longer than 14 days (E76A), the costs per day must be negotiated separately between SHI and the respective hospital and will vary accordingly. While the InEK provides detailed data on the length of stay for all three DRG categories, but cost data only for E76B and E76C [9], the various reimbursements to the hospitals for E76A patients are collected by the German Hospital Association (Deutsche Krankenhausgesellschaft e.V.) [10]. To ensure that exclusively new cases were involved, we only used health insurance data from patients for whom TB disease was reported as the main diagnosis. The case numbers used for our overall cost calculation are thus lower than the total number reported. Per-day drug costs are calculated based on the maximum recommended dose for each drug being administered. Where several alternatives exist for the same effective substance, the lowest-cost presentation is chosen and costed according to the 2023 issue of the Rote Liste® (red list).

As portions from routine surveillance data and fixed or pre-weighted costs are used in our non-probabilistic model, confidence intervals are not provided. Although numerous side effects can occur throughout therapy, costs for adjuvant medication do not apply because data on treating and monitoring these side effects is not routinely collected. Costs are reported in 2023-Euros (EUR) and considered only over 6 months, therefore a need for discounting did not arise.

## 4 Diagnostics and therapy of active non-MDR-TB

In Germany, the responsibility for management of the diagnosis and therapy for primary outpatient treatment or for secondary outpatient treatment following initial hospitalization of adults lies with the general practitioner throughout the course of the disease. Usually, the general practitioner serves as a “door opener” and will either refer the patient directly to the hospital where the patient will start his/her diagnostic procedures and therapy as an inpatient or, alternatively to a pulmonologist in private practice. The latter, in turn, decides whether the patient should be cared for primarily as an outpatient. If an initial hospital stay is required, the patient later returns to the pulmonologist for post-stay care. According to the guidelines of the DZK [3], the therapy for pulmonary TB lasts at least 6 months. In Germany, 11.4 % of all culturally verified cases of TB are resistant to at least one of the first-line drugs [6], so the quadruple combination with INH, RIF, pyrazinamide (PZA) and ethambutol (E) is recommended. After an “initial phase” of two months, patients with fully sensitive strains are treated with INH and RIF for a further 4 months in the subsequent continuity phase. Table 1. lists the corresponding medication costs. Table 2 includes the outpatient remuneration for the following procedures and controls:

1. In patients with suspected pulmonary TB, an interferon-gamma release assay (IGRA) and a chest X-ray are generally the first steps. Following referral to a pulmonologist, these are followed by bacteriological confirmation − usually from sputum or bronchoalveolar lavage following referral to a pneumonologist. This may include microscopic verification of acid-fast bacilli (indicator of contagiousness) and/or culture (pathogen verification) together with a NAAT assay for rapid identification of the M. tuberculosis complex or of MDR, e.g. by the WHO-endorsed Xpert MTB/RIF Ultra® (Cepheid).
2. For the purpose of monitoring, three expectorated sputum samples (for the larger yield) are collected on separate days for microscopy in patients with suspected pulmonary TB in the initial phase and then every 4 weeks until negative results are obtained, i.e. at least 5 times for a presumed conversion within 8 weeks. Cultures are made in the initial phase (positive culture results followed by resistance testing), then after 4 and 8 weeks (sputum conversion should have occurred by that time) and then once more towards the end of the therapy (verification of successful therapy according to WHO criteria).
3. Blood work: A full blood profile is necessary before therapy starts, together with kidney retention values (creatinine, urea) and liver values (ALT, AST, bilirubin, GGT). [Liver values are required as alcohol abuse parameter; they also allow for differentiation between INH-induced hepatitis and RIF-induced cholangitis in case this value increases as a result of the medication] It is also advisable to determine uric acid level in adults (as compliance parameter because an increase under PZA is almost certain), together with hepatitis serology (HBs-Ag, Anti-HBc), as well as HIV serology. Liver values are controlled 2–4  weeks after starting the therapy, and every 4  weeks thereafter (bilirubin is not checked if there are no anomalies in the liver parameters). Uric acid values are checked only every 4  weeks until PZA is stopped, i.e. altogether only twice in standard therapy if prior hyperuricemia was not reported. Blood tests and renal retention parameters are checked every month through to the end of the therapy, together with checks of the liver values.
4. Ophthalmic examination: Under E, before therapy starts and usually every 4  weeks, in order to exclude optic neuropathy.
5. Chest X-rays: in the initial phase, after 4  weeks (to control whether the TB foci start decreasing under treatment), and after 8  weeks (end of the initial therapy: success assessment). Additional X-ray check-ups in the 4th and 6th month are sufficient, followed by check-ups 6, 12 and 24 months after the end of treatment.

**Table 1:** SHI costs for outpatient therapy of active pulmonary TB: medication.

Medication^1^			
	Costs/day (rounded) in EUR	Costs in EUR^2^	Costs post-hospital stay (minus 31 days of hospital stay)
RIF^3^	2.89	520.2	430.61 (149 days)
INH^4^	0.44	79.2	65.56 (149 days)
E^5^	1.76	105.6	51.04 (29 days)
PZA^6^	1.65	99	47.85 (29 days)
**−**	−	804	595.06

1 Based always on the smallest pack available for the necessary minimum period of treatment. The daily therapy costs are calculated from the quotient between the intake quantity stated in the dosing instructions and the pack quantity and multiplied by the number of treatment days.

2 Based on an average treatment period of 180 days for isoniazid and rifampicin; 60 days are taken for pyrazinamide and ethambutol using the recommended maximum dose in each case.

3 Eremfat 600 Tbl., one tablet taken once a day as instructed (maximum dose 600 mg).

4 Isozid comp 300 Tbl., one tablet taken once a day as instructed (maximum dose 300 mg).

5 EMB FATOL 400 Tbl. four tablets taken once a day. (maximum dose 1600 mg).

6 Pyrazinamid JENAPHARM 500 Tbl., five tablets taken once a day (maximum dose 2500 mg).

**Table 2:** SHI costs for outpatient therapy of active pulmonary TB: medical services.

Medical services	Individual payment (EUR)	Frequency	Payment (EUR)	Posthospital payment (EUR)
General practitioner				
1 Flat rate coverage; irrespective of number of visits by patients per quarter (GOP 03000) from the age of 19 until the age of 54	13.60	5 [quarters]	68	4 [quarters]: 54.4
2 Detailed conversation (GOP 04230)	15.28	2	30.56	1 [1st quarter]: 15.28
3 Retainer fee (general practitioner), once per quarter (GOP 03040)	16.47	5 [quarters]	82.35	4 [quarters]: 65.88
Pneumological diagnostics				
4 Pneumological consultation; 6 to 59. years of age (GOP 13641)	24.82	5 [quarters]	124.1	4 [quarters]: 99.28
5 Surcharge (pneumologist), once per quarter (GOP 13644)	4.89	5 [quarters]	24.45	4 [quarters]: 19.56
6 Bronchoscopy (GOP 13662)	136.29	1	136.29	−
7 Pneumologist BAL (GOP 13663), additional fee on GOP 13662	26.73	1	26.73	−
Methods				
8 IGRA testing (GOP 50112 following GOP 0220)	58	1	58	−
9 ECG (GOP 27320); cannot be charged separately	−	−	−	−
10 X-ray in two planes (GOP 34241); a consultation cannot be charged	17.42	8	139.36	104.52 (6 X-rays following initial hospitalization)
11 For ethambutol treatment: ophthalmologic consultation (GOP 06211 for insured patients aged between the 6 and 59 years)	13.96	3	13.96 (only be charged as flat once per quarter)	−
Microbiology				
12 Microscopy test for mycobacteria (GOP 32176)	5.20	5	26	15.6
13 NAAT (GOP 32825)	61.40	1	61.40	−
14 Culture test for mycobacteria (GOP 32747) per material	34.90	4	139.60	104.7
15 Differentiation of mycobacteria (GOP 32764) if positive	28.40	1	28.40	−
16 Molecular fast resistance testing (GOP 50110 if NAAT or microscopy is positive)	82.03	1	82.03	−
17 Resistance definition (GOP 32770) per mycobacteria type	39.50 (maximum value)	1	39.50	−
Laboratory investigation				
18 HIV serology, immune-assay combination test (GOP 32575)	4.45	1	4.45	−
19 Anti-HBc (GOP 32614)	5.90	1	5.90	−
20 HBs-Ag (GOP 32781)	5.50	1	5.50	−
21 Chloride (GOP 32084)	0.25	8	2	1.25 (5 * 0.25)
22 Sodium (GOP 32083)	0.25	8	2	1.25
23 Potassium (GOP 32081)	0.25	8	2	1.25
24 Calcium (GOP 32082)	0.25	8	2	1.25
25 Creatinine, Jaffe method (GOP 32066)	0.25	8	2	1.25
26 Uric acid (GOP 32964)	0,25	2	0.5	0.25
27 Urea (GOP 32065)	0.25	8	2	1.25
28 Blood count (GOP 32122)	1.10	8	8.80	5.50
29 Bilirubin total (GOP 32058)	0.25	8	2	1.25
30 Gamma-glutamyl transferase (GOP 32071)	0.25	8	2	1.25
31 Glutamate-oxaloacetate transaminase (GOP 32069)	0.25	8	2	1.25
32 Glutamate-pyruvate transaminase (GOP 32070)	0.25	8	2	1.25

Anti-HBc = Hepatitis B core antibody; BAL=bronchoalveolar lavage;

HBs–Ag = surface antigen of the hepatitis B virus; NAAT=Nucleic Acid Amplification Test.

## 5 Treatment of latent TB

The intensive work done in the 2000s on refining preventative chemotherapy to eliminate latent tuberculosis has resulted in shorter-course, less toxic approaches, the cornerstone of which is RIF. The latest recommendations therefore stipulate that in adults, RIF (10 mg/kg body weight once daily orally, maximum daily dose 600 mg) be given as preventive therapy for 4 months, or that RIF and INH be taken in combination (dosage analogous to the respective monotherapy) for 3 months. For now, straight INH treatment (5 mg/kg/KG once daily per os, maximum daily dose 300 mg, for 9 months) has not been excluded. Given the advantages of the newly confirmed, shorter-term regimens, however, it can be expected that its use will in practice be discontinued.

Contact persons undergoing RIF treatment (with or without combination with INH) need to visit a physician every month for examination (including a brief checking for signs of hepatitis, such as dark urine and icterus) and be closely monitored for symptoms of hepatic disease. The reason is that RIF alone may cause hepatoxic side effects in a small percentage of people (RR 0.12; 95 % CI 0.05 to 0.30), and that the risk of heptotocicity may potentially increase when adding INH to RIF for 3 months [11], [12].

Laboratory analyses include periodic liver values (ALT, AST, GGT), renal retention parameters (creatine, urea), and blood count at baseline before starting treatment and then every four weeks through the end of the therapy (Table 4). The standard bilirubin check need not be repeated if there are no anomalies in the initial liver parameters. HIV serology should be determined as the presence of an HIV infection will require prolonged treatment [13]. Asymptomatic serum AST increases are expected and usually do not indicate that treatment need be stopped.

Close contacts undergo chest radiography not only before beginning TPT (an act that is generally performed by the public health service) but also at the end (the cost of which our analysis includes).

## 6 Results

### 6.1 Inpatient cost (direct hospital costs)

In 2022, 308 TB patients belonging to the DRG category E76C were treated in hospitals for, on average, 6.3 days (SD±3.3 days), and 301 TB patients belonging to DRG category E76B were treated, on average, for 6.5 days (SD±4.1 days). Only 2442 TB patients, were assigned to category E76A, i.e. hospitalized at least 14 days, with an average of 38.2 days (SD±27.5); the mean reimbursement for those patients was 6454.52 EUR (SD±3165.79 EUR) [10]. Thus, after subtracting the 184 MDR-/RR-TB cases from the total of 2442 E76A TB patients, the weighted mean costs for non-MDR-TB patients treated in German hospitals can be calculated as follows: (308 patients * 4331.81 EUR [E76C]) + (301 patients * 5295.76 EUR [E76B]) + (2258 patients * 6454.52 EUR (SD±3165.79 EUR) [E76A]) divided by the total of 2867 patients, amounting to 6137.78 EUR (SD±2809.71 EUR).

The mean treatment time in hospital per person was calculated for all 3 DRG categories by multiplying the respective mean treatment days by the number of patients and then dividing by the total number of hospitalized patients [308 * (6.3 ± 3.3 days)] + [301 * (6.5 ± 4.1 days)] + [2258 * (38.2 ± 27.5 days)] / 2867], resulting in 31.44 ± 24.47 days.

### 6.2 Outpatient costs in private practice

Outpatient costs at the expense of the SHI comprise costs of first-line drugs for 6 months (for primary outpatients) or 6 months minus 31 days (for patients following hospitalization) and the costs of services and providers for diagnosing and monitoring as suggested above according to the SHI scheme [7]. After adding medication to the costs described in Table 1 and the reimbursements for the 32 positions listed in Table 2, the costs for primary outpatient treatment costs for adults are 1929.88 EUR and the outpatient costs for those adults after the hospital stay are 1092.53 EUR (positions 1–5, 10, 12, 14, 21–32).

### 6.3 Weighted combined inpatient/outpatient TB costs

Hospital treatment was provided in 83.9 % of all TB cases [4], so in adults the weighted costs for a standard TB case are [(6137.78 EUR (SD±2809.71 EUR) + 1092.53 EUR [outpatient treatment post-hospital]) x 0.839]) + (1929.88 EUR [primary outpatient treatment] x 0.161), i.e. 6376.95 EUR (SD±2357.35 EUR).

### 6.4 TPT costs

For a 4-month RIF preventive treatment, the drug costs of RIF for 4 months are 373.90 EUR (Table 3) and the general doctoŕs fees and laboratory costs (positions 1–11) in Table 4 amount to 91.66 EUR, i.e. summing up to total costs of 465.56 EUR. The total costs of the 3-months combined RIF/INH treatment is lower (89.31 EUR plus 333.43 EUR), amounting to 422.65 EUR.

**Table 3:** SHI costs of TPT: medication.

Medication			
	Costs per package (cheapest option in EUR)	Duration (days)	Costs (in EUR)
Option 1: RIF/INH combined (3 months)			
RIF	100 Tbl 600 mg: 289.06	90	289.06
INH^1^	100 Tbl 300 mg: 44.28	90	44.28
			333.34
Option 2: RIF (4 months)			
RIF	100 Tbl 600 mg: 289.06	100	289.06
	10 Tbl 600 mg: 42.42	10	42.42
	10 Tbl 600 mg: 42.42	10	42.42
			373.90

1 Isozid comp 300 mg Tbl 100 (N3), one tablet taken once a day, is the cheapest option. The costs for the remaining 10 tablets have to be incurred even if they are no longer taken.

**Table 4:** SHI costs of TPT: medical services.

Medical services	Individual payment (in EUR)	Frequency	Payment (in EUR)^1^
General practitioner			
1 Flat rate coverage; irrespective of number of visits by patients per quarter (GOP 03000) from the age of 19 until the age of 54)	13..0	2	27.20
2 Retainer fee (general practitioner), once per quarter (GOP 03040)	16,47	2	32.94
Methods			
3 X-ray in two planes (GOP 34241); a consultation cannot be charged	17.42	1	17.42
Laboratory tests			
4 HIV serology, immune-assay combination test (GOP 32575)	4.45	1	4.45
5 Creatinine, Jaffe method (GOP 32066)	0.2	4 (3)	1 (0.75)
6 Urea (GOP 32065)	0.25	4 (3)	1 (0.75)
7 Blood count (GOP 32122)	1.1	4 (3)	4.4 (3.3)
8 Bilirubin total (GOP 32058)	0.25	1	0.25
9 Gamma-glutamyl transferase (GOP 32071)	0.25	4 (3)	1 (0.75)
10 Glutamate-oxaloacetate transaminase (GOP 32069)	0.25	4 (3)	1 (0.75
11 Glutamate-pyruvate transaminase (GOP 32070)	0.25	4 (3)	1 (0.75)

1 Payment for the 3-month combined RIF/INH treatment in brackets.

## 7 Discussion

As the new recommendations for treating and preventing TB have only recently been published in Germany, we took the opportunity to update the cost data for treating infectious pulmonary TB disease and for TPT in freshly infected contact persons. Compared to the mean combined inpatient and outpatient costs for pulmonary non-MDR-TB in adults of our benchmark study published 4 years ago [14], the calculated mean treatment costs covered by the German SHI have markedly decreased, i.e. by rounded 27 % (6377 EUR vs. 8756 EUR). First, this is thanks to a decrease in hospitalization rates for TB patients of 5.6 %, from 89.5 % in 2018 to 83.9 % in 2022, thus increasingly shifting the responsibility for monitoring and treating TB in Germany to the generally less expensive outpatient sector. Second, given hospital treatment, the per-day reimbursement negotiated between SHI and the few hospitals in Germany that are still willing to treat TB patients has decreased from 326 EUR to 231 EUR over the same period (see Table 5), although the mean length of hospital stay per TB case increased from 25 to 31 days (rounded).

**Table 5:** Per-day reimbursement for German TB patients hospitalized at least 14 days.

Year	DRG	Cases (No.)	Days (Sum)	Reimbursement by SHI [10]			
				Minimum (in EUR)	Mean (in EUR)	Maximum (in EUR)	Standard Deviation (in EUR)
2019	E76A	2906	87,431	216.92	325.84	554.37	47.81
2020	E76A	2532	75,557	106.20	227.41	1999.85	75.33
2021	E76A	2246	68,119	117.37	225.61	495.98	68.15
2022	E76A	2278	70,679	101.80	231.34	524.93	68.87

Our calculation of costs for treating the active pulmonary TB disease has several limitations. Our hospital cost data consider resistance or intolerance of some patients to any of the four antituberculosis drugs, especially against INH. Data on alternative regimens that may have been more costly, e.g. by adding fluoroquinolones, were not, however, available for the post-hospital period, and also not available for the 16.1 percent of patients who were primarily treated in private practice. As such, our updated analysis may underestimate the real costs of non-MDR-TB and represents only the minimum of costs that may be incurred in Germany for diagnosing and treating TB disease in Germany.

Nevertheless, the fall in cost of standard treatment for active pulmonary TB over the past 4 years contrasts with the sharply risen costs of the short-term TPT to 466 EUR (RIF solely over 4 months) per person and 423 EUR (RIF combined with INH over 3 months). In the past, TPT was based entirely on INH, the cost of which is almost negligible. Whereas bringing costly RIF into the program significantly improves the overall efficacy of TPT, its now elevated cost requires that it be applied more carefully than in the past. The selection of contact persons who may be subjected to IGRA testing for latent TB and, if they are tested positive, will be offered TPT later on, must be strictly carried out. From a test-theoretical point of view, only contact persons of infectious pulmonary TB cases should be considered for TPT when they may have been a truly “close” contact rather than a casual contact, so as to increase the prevalence of latent TB among persons being tested and thus the positive predictive value of a positive IGRA test. To reduce the number of contacts who have a positive test but in fact are not at risk of progressing to a disease state, the 2023 update of the German recommendations on contact tracing [3] contain a rule to require 8-hours cumulative exposure time as a prerequisite for environmental IGRA testing, as long as there was no evidence of direct “face-to-face” contact with a coughing index case. Therefore, careful collection of actual contact times rather than any exposure towards an infectious TB patient could making contact investigations, and subsequently conducted TPT, more effective.

## 8 Conclusions

Whilst the costs for active non-MDR-TB treatment in Germany have clearly decreased thanks to stronger engagement of the private practice sector and lower per-day reimbursement rates for hospitals, the comparatively high costs of short-course TPT have surprisingly significant economic impact. Diligent selection of close contacts persons of infectious TB cases to receive IGRA testing for latent TB is recommended to minimize the number of those persons who unnecessarily receive treatment and thus to make TPT overall more cost-effective. Therefore, particular attention should be paid not only to the duration of the contact period but to the intensity of contact as well.

## Ethical statement

This cost study contains freely accessible or published data and does not refer to specific patients. Approval by an ethics committee was therefore not required.

## CRediT authorship contribution statement

**Roland Diel:** Writing – original draft, Methodology, Formal analysis, Conceptualization. **Albert Nienhaus:** Writing – review & editing, Conceptualization.

## Declaration of competing interest

The authors declare that they have no known competing financial interests or personal relationships that could have appeared to influence the work reported in this paper.
